# Increased Plasma Endogenous Ethanol Correlates with and Discriminates Against Microvascular Complications in Type-2 Diabetic Traditionally Alcohol-Abstinent Saudi Patients: A Biomarker Cross-Sectional Analysis

**DOI:** 10.3390/metabo16100732

**Published:** 2026-09-29

**Authors:** Basil Mohammed Alomair, Alaa Abdelhamid Mohamed, Abrar Zayed Ashalan, Dala Sultan Alruwaili, Modi Moisheer Almoisheer, Reem Farhan Alruwaili, Rimas Hamdan Alazmi, Hanan Qasem Alruwaili, Abdulrahman Abdulwahab Alduriwish, Tarek Hassan El-Metwally

**Affiliations:** 1 Department of Internal Medicine, College of Medicine, Jouf University, Sakaka 72388, Saudi Arabia; dr-aaad@ju.edu.sa; 2 Biochemistry Division, Department of Pathology, College of Medicine, Jouf University, Sakaka 72388, Saudi Arabia; alaaali@ju.edu.sa; 3 Department of Medical Biochemistry, Faculty of Medicine, Beni-Suef University, Beni-Suef 62521, Egypt; 4 College of Medicine, Jouf University, Sakaka 72388, Saudi Arabia; ar.xx04@gmail.com (A.Z.A.); dlalrwyly6@gmail.com (D.S.A.); modialmoisheer@gmail.com (M.M.A.); reemalwili@gmail.com (R.F.A.); rimasalazmi6@gmail.com (R.H.A.); 5 The Research Laboratory, College of Medicine, Jouf University, Sakaka 72388, Saudi Arabia; hqalruwaili@ju.edu.sa; 6 Department of Medical Biochemistry and Molecular Biology, Faculty of Medicine, Assiut University, Assiut 71526, Egypt

**Keywords:** endogenous ethanol, type-2 diabetes, glycemic control, risk factor, diabetes complications, microbiome dysbiosis

## Abstract

**Background/Objectives**: Endogenously produced ethanol (EndoEth) from a dysbiotic microbiome is an under-recognized pathogenic factor in metabolic diseases, including type-2 diabetes (T2DM). EndoEth is any systemically detectable alcohol in abstinent individuals. We aimed to assess EndoEth changes, its correlation with patients’ characteristics, metabolic indices, the disease outcomes, and its biomarker and independent risk factor characteristics as compared to healthy controls. **Methods**: EndoEth was cross-sectionally assayed using a specific enzymatic colorimetric method in 125 abstinent T2DM patients and 55 matching healthy controls. Its associations with glycemic control [fasting blood glucose (FBG), HbA1c, and triglyceride–glucose insulin resistance (TyG-IR) index], lipid profile [total, LDL-(LDL-C), and HDL-cholesterol (HDL-C), and triglycerides (TGs)], and the disease duration, treatment and microvascular complication profile (ophthalmopathy, neuropathy and nephropathy) were assessed. Its biomarker ability was assessed using the area under the ROC curve (AUC). **Results**: Significantly higher levels of EndoEth were detected in T2DM patients. EndoEth had significant associations with disease duration, complications particularly ophthalmopathy, insulin treatment, fasting blood glucose, TyG-IR index, and plasma triglycerides. Complications were also significantly associated with age, male gender, disease duration, FBG and total cholesterol. ROC curve analysis showed acceptable moderate ability of plasma EndoEth to discriminate against T2DM complications (AUC mean ± standard error = 0.766 ± 0.047, *p* = 0.001, and at a cut-off ≥ 0.016 µmol/mL, sensitivity = 75.7% and specificity = 61.0%). **Conclusions**: EndoEth is higher in abstinent T2DM patients than in healthy controls, and is correlated with disease duration, insulin resistance and treatment and complications, particularly for ophthalmopathy. It had a moderate biomarker performance (AUC = 0.766) in discriminating against diabetic complications.

## 1. Introduction

Endogenous ethanol (EndoEth) is the systemically detectable amounts of ethanol produced by the fermentation of the available substrates by the gut, oral and genito-urinary microbiome upon alcohol abstinence. Increases in EndoEth synthesis are seen in bacterial/fungal overgrowth in oral, gut, bladder and urogenital fermentation syndromes (aka, auto-brewery syndromes; ABS). The under-recognition of fermentation syndromes, as under-diagnosed medical conditions, has not only clinical consequences but also adverse social and legal sequelae for patients. Monitoring the blood alcohol is the gold-standard diagnostic approach [1,2]. Because of the liver’s efficient clearance of ethanol by oxidation (95%), and excretion in urine and breath (5%), a detectable level in body fluids indicates a rate of EndoEth biosynthesis that exceeds 6–8 g/hour. Hence, it is not surprising to find that post-prandial portal blood EndoEth level is >100-fold that of the peripheral blood. Microbiome dysbiosis, a disruption in the normal balance of microbial communities, is associated with a range of systemic diseases, encompassing gastrointestinal, metabolic, neurological and psychiatric, cardiovascular, and immune-related disorders. Other than disrupting integrity of the mucosal barrier, major dysbiosis pathogenic effectors include the produced metabolites, including ethanol, with unwanted local mucosal and systemic effects [3,4,5,6].

Microbiome dysbiosis alters the rate of production and hence the blood level of the absorbed EndoEth, potentially presenting a pathogenically aggravating factor. Diabetes and obesity are both associated with microbial imbalance. The extent of microbiome dysbiosis and its sequelae are expected to be more pathologically significant in diabetic patients. In these patients, elevated EndoEth production has been observed, indicating a potential pathway through which microbiome-related ethanol production could exacerbate inflammatory and disease prognosis [7,8,9,10]. Microbiome dysbiosis and increased EndoEth production were previously associated with hyperglycemia, obesity, and uncontrolled diabetes [8,9,11]. Increases in EndoEth indicate increased fermentation and/or diminished ability to dispose of alcohol. Other than its consumption by gut microbes themselves and exhalation, alcohol is oxidized into acetaldehyde mainly by alcohol dehydrogenase, then to acetate by aldehyde dehydrogenase, chiefly in the liver. Fasting ethanol levels are positively associated with insulin resistance and were significantly higher in children with non-alcoholic fatty liver disease [(NAFLD), recently reclassified as metabolic dysfunction-associated steatotic liver disease (MASLD)] than in controls. Insulin-dependent impairments of alcohol dehydrogenase activity in liver tissue were implicated, rather than from increased EndoEth synthesis [1,12]. Obesity and diabetes are among the prominent risk factors for ABS. Carbohydrate-rich food challenge test, or routine intravenous or 200 g oral glucose tolerance tests, is a part of the differential diagnosis of the syndrome, where increases in blood and breath alcohol correlate to blood glucose changes [13,14,15,16].

Studying the relationship between diabetes and EndoEth levels, as a surrogate biomarker for dysbiosis, holds the promise of furthering our understanding of the complex pathophysiology of the disease and its prognosis, ideally in an ethanol-abstaining population with prevailing obesity and T2DM, energy-rich food, sedentary lifestyle, and environmental and genetic background.

## 2. Materials and Methods

### 2.1. Study Design and Setting

We evaluated the variations in and associations between fasting plasma EndEth levels and glycemic control indices, and complication profiles in T2DM patients in a cross-sectional design. The study enrolled hospital-diagnosed patients followed-up at the diabetes outpatient clinics of the Diabetes Center of King Abdulaziz Specialized Hospitals in Sakaka, Saudi Arabia.

### 2.2. Study Participants and Sampling

The required sample size was calculated using OpenEpi online software (version 3.01, https://openepi.com/) for a cross-sectional case–control comparison of a continuous outcome (plasma endogenous ethanol concentration). Assuming a two-sided significance level (α) of 0.05, a power (1-β) of 0.80, and based on a pilot study from a comparable population [17] reporting a mean difference in EndoEth of approximately 0.04 µmol/mL between healthy abstinent individuals and those with metabolic disease, with a common standard deviation of 0.06 µmol/mL, the calculated effect size (Cohen’s d) was 0.67. The minimum required sample size was 36 participants per group. To account for the known prevalence of type-2 diabetes in the Sakaka population (~25%) and a potential 15% dropout or exclusion rate, we targeted a total of 180 participants. The final sequential enrolment of the 125 consented T2DM clinically confirmed hospital-based diagnosed patients and age-, sex distribution-, and BMI-matching of the 55 healthy controls exceeded the minimum requirement, providing adequate statistical power for the primary and secondary analyses. Alcohol abstinence relied on the self-reported history of participants. Detection of measurable levels of EndoEth was expected to be >21%, as previously reported for healthy adult alcohol-abstinent residents of Saudi Arabia [17].

We excluded patients on/exposed to alcohol at any amount (as alcoholic beverages, or contained in a medication, a medicinal preparation, mouthwash or colognes and hand sanitizers), those with diabetic severe complications (current ketoacidosis, diabetic foot, and gangrene), short gut syndrome and inflammatory bowel diseases, chronic kidney and liver disease, autoimmune diseases (including T1DM), those who had undergone bariatric surgeries, those on hemo-diluting fluid therapy, all types of cancer, those on antibiotics in the previous week, those who were immuno-compromised, inflammatory affections unrelated to T2DM, pregnancy, and immobility. None of our participants exhibited diagnostic criteria of ABS or were previously diagnosed with it or with small intestinal bacterial overgrowth (SIBO).

### 2.3. Investigations and Data Collection

We collected the following relevant de-identified sociodemographic, anthropometric laboratory and clinical data from medical records following the last follow-up visit: Sex, age, and body mass index, disease duration, macrovascular complications (cardiac and stroke), microvascular complications (ophthalmopathy, nephropathy, or neuropathy), type of the antidiabetic treatment (naïve and/or diet control, metformin as a first-line hypoglycemic drug, other hypoglycemics ± metformin, or, insulin ± hypoglycemics), fasting metabolic biomarkers [lipid profile (total, low density lipoprotein (LDL)- and high density lipoprotein (HDL)-cholesterol, and triglycerides), fasting glucose, hemoglobin A1c (HbA1c)], and total leukocytic (WBC) count. We used the triglyceride–glucose index as a non-insulin-based surrogate marker of the insulin resistance (TyG-IR). This index was calculated as Ln [(triglycerides, mg/dL × glucose, mg/dL)/2] [18]. Treatment was scored as: treatment naïve/diet control = 0, metformin = 1, hypoglycemics including metformin = 2, and insulin ± hypoglycemics = 3.

EndoEth blood levels were determined using a specific quantitative colorimetric enzyme assay, according to the manufacturer’s instructions (Cat# E-BC-K891-M, Elabscience, Houston, TX, USA). The assay has a recovery rate of 96%, inter-assay coefficient of variation (CV) of 5%, intra-assay CV of 3.5%, and a lower limit of detection (LoD) of 0.27 μmol/mL. It employs ethanol dehydrogenase and NAD to produce acetaldehyde and NADH that, in the presence of 1-Methoxyphenazine, converts 2-(2-methoxy-4nitrophenyl)-3-(4-nitrophenyl)-5-(2,4-disulfophenyl)-2H-tetrazolium into a yellow-colored formazan measurable at 450 nm. For collecting overnight fasting plasma, peripheral blood sample was aseptically collected on EDTA from the antecubital vein after isopropanol swabbing. Samples were centrifuged for 20 min at 4 °C and 1000× *g*. Recovered plasma was stored at −60 °C until assayed. Participants with EndoEth contents lower than the LoD were presented as 0.0 content.

### 2.4. Ethical Consideration

The study was bioethically approval by the Permanent Research Ethics Committee of Jouf University, Sakaka, Saudi Arabia (Approval# 11556 on 4 April 2026). Each participant signed a written informed consent. Access to the raw and analyzed data was confined to the biostatistician, and members of the study team and data were saved in password-protected documents. The study adhered to the tenets of the 2024-revised Declaration of Helsinki.

### 2.5. Statistical Analysis Plan

Data analysis and presentation were done using IBM SPSS Statistics for Windows (version 27.0). The Shapiro–Wilk test was used to confirm distribution of continuous variables that are expressed as mean ± standard deviation or standard error (range), or median ± IQR (range). The categorical data are presented as frequencies and percentages (n, %). The chi-squared test was used for group comparisons of categorical variables. The independent samples t-test was used for group comparisons for normally distributed variables, and the Mann–Whitney U test and Kruskal–Wallis test for non-normally distributed parameters. Spearman’s correlation coefficient assessed the associations between plasma EndoEth levels and other continuous parameters. The multivariable logistic regression model was used to identify independent risk factors associated with diabetic complications, with the results reported as adjusted odds ratios (ORs) and 95% confidence intervals (CIs). Receiver Operating Characteristic (ROC) curve analysis was used to determine the diagnostic performance of EndoEth. The optimal cut-off value was determined using Youden’s index. A two-tailed *p* value < 0.05 was considered statistically significant.

## 3. Results

Baseline demographic and anthropometric characteristics of the healthy controls were not significantly different from T2DM patients for sex distribution [female/male n (%); 27 (49.1%)/28 (50.9%)], age [Mean ± SD (Range); 54.2 ± 6.00 (43–68)], and, BMI [Mean ± SD (Range); 29.19 ± 3.87 (19.81–43)]. Liver function indices were within the normal range for all participants (alanine and aspartate transaminases, alkaline phosphatase, γ-glutamyl-transferase, total and direct bilirubin, and total proteins and albumin). The disease duration ranged from 0.5 to 32 years, and 29.6% of the patients had microvascular complications. One myocardial infarction and two stroke cases of macrovascular complication could not be statistically represented further. Ophthalmopathy was the majority (n = 21), followed by neuropathy (n = 15), then nephropathy (n = 3); some patients had more than one complication. The majority of patients were on metformin as a first-line hypoglycemic drug (39.2%), followed by insulin-containing treatment regimen (36.8%) (Table 1).

Table 2 presents the comparison of the basic laboratory variables and plasma EndoEth content of healthy controls vs. T2DM patients. All of them showed highly significant differences. Stratifying our patients or controls for sex did not present significant gender-dependent differences in EndoEth levels (*p* = 0.566 and *p* = 0.875, respectively). However, comparing male or female controls to male or female patients, like the case in which they were compared as whole groups (*p* < 0.001), showed highly significant increases in patients (*p* = 0.002 and *p* = 0.003, respectively).

Table 3 shows that stratifying T2DM patients for presence or absence of complications (*p* < 0.001) and type of complications reveals that EndoEth levels were significantly higher in patients with ophthalmopathy (n = 21; *p* = 0.001) and nephropathy (n = 3; *p* < 0.001), but not in those with neuropathy (*p* = 0.309). Since there were only three nephropathy patients, this finding should be taken very cautiously. EndoEth levels also showed progressive increases in treatment-naïve patients, through those on hypoglycemics, to those on insulin-containing regimen (*p* = 0.001).

Spearman correlation analysis for EndoEth with different parameters among patients is presented in Table 4. It showed significant positive correlation with each disease duration (r = 0.298 and *p* = 0.001) with a moderate effect size, FBG (r = 0.193 and *p* < 0.040) with a small effect size, triglycerides (r = 0.303 and *p* = 0.001) with a moderate effect size, and TyG-IR index (r = 0.274 and *p* = 0.003) with a moderate effect size.

The association between the different investigations and presence of complication among T2DM patients is presented in Table 5. Significant association was evident with age advancement (*p* = 0.033), male gender (*p* = 0.015), longer disease duration (*p* < 0.001), higher fasting blood glucose (*p* = 0.008), higher total cholesterol (*p* < 0.001), higher LDL-C (*p* = 0.033), and higher EndoEth (*p* < 0.001).

Table 6 presents the results of the multivariable logistic regression model for independent risk factors for the development of diabetic complications among T2DM patients. Total cholesterol (*p* < 0.001) and LDL-C (*p* < 0.001) were independent predictors of the occurrence of complication. Because TC and LDL-C represent closely related lipid measures, sensitivity analyses were additionally performed using separate models containing TC or LDL-C. The models found that EndoEth was significantly associated with complications when adjusted separately for either TC or LDL-C, but the association becomes nonsignificant when both closely related lipid measures were entered simultaneously. After adjustment for TC and LDL-C, each 0.01 µmol/mL increase in EndoEth (which was divided by 0.01, so that OR represents the change in odds for every 0.01 µmol/mL increase in it) was associated with approximately 6.1% higher odds of complications, but this association was not statistically significant. TC and LDL-C were moderately correlated, but the VIF/tolerance diagnostics did not indicate problematic multi-collinearity.

**Figure 1:**
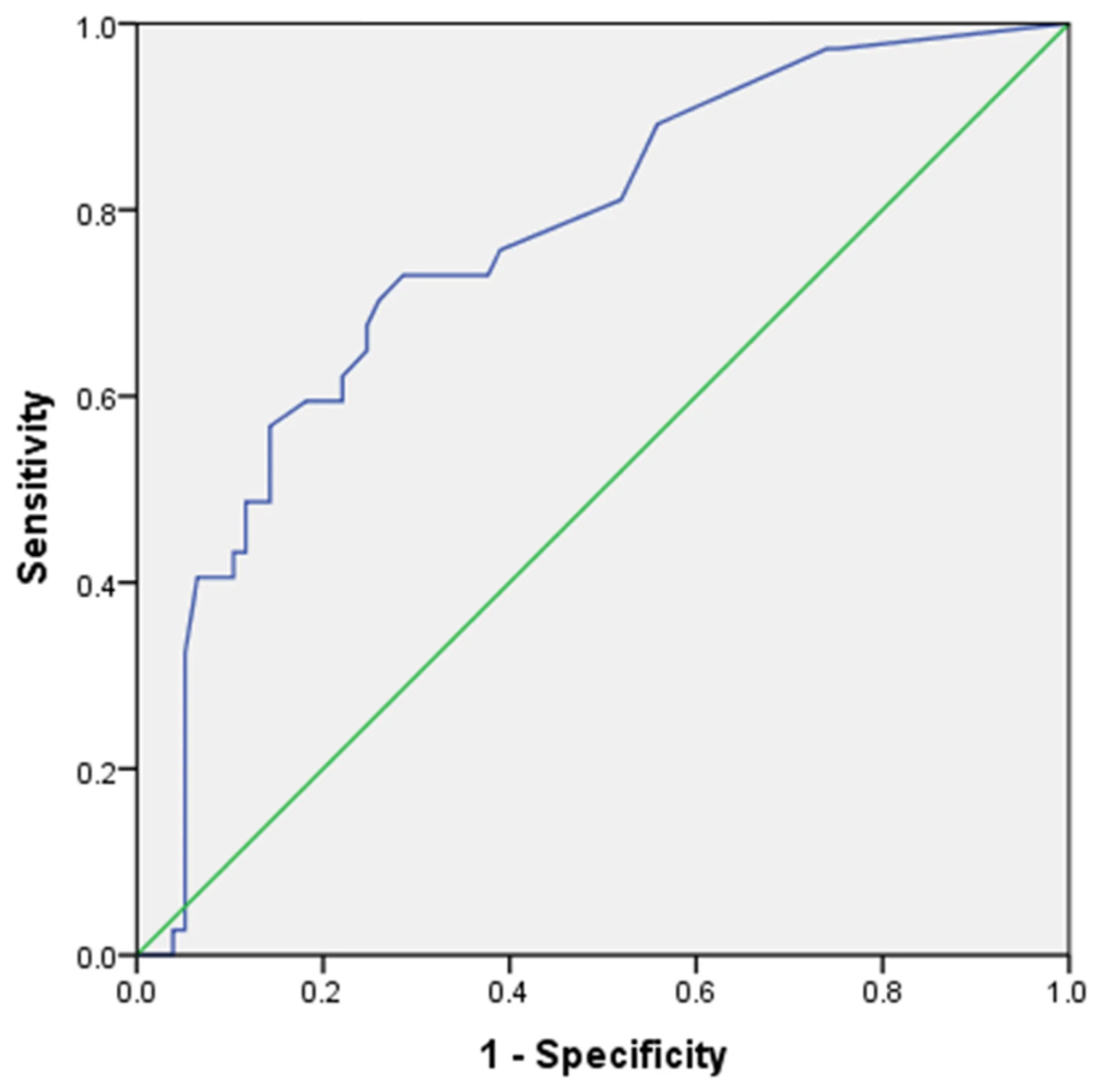
Area under the ROC curve (AUC) analysis of the biomarker potential of plasma endogenous ethanol to assess the discriminatory ability of EndoEth for the occurrence of diabetic complications among T2DM patients. Diagonal segments are produced by ties. AUC mean ± standard error = 0.766 ± 0.047, *p* = 0.001, and asymptotic 95% confidence intervals had lower and upper bounds of 0.673 and 0.858. At a cut-off point of ≥0.016 µmol/mL (determined using the Youden index J = 0.516), sensitivity = 75.7% and the calculated specificity = 61.0%.

Receiver Operating Characteristic (ROC) analysis was performed among the diabetes cases to assess the discriminatory ability of EndoEth for the occurrence of diabetic complications (Figure 1). The area under the ROC curve (AUC) was 0.766 (standard error = 0.047; 95% CI: 0.673–0.858; *p* < 0.001), indicating fair-to-good discriminatory ability of EndoEth to distinguish diabetes patients with diabetic complications from those without complications. The AUC was significantly greater than 0.50, demonstrating that the discriminatory performance of EndoEth was significantly better than chance. The ROC coordinates demonstrated a trade-off between sensitivity and specificity across increasing EndoEth thresholds. At an EndoEth threshold of 0.016 µmol/mL, the sensitivity was 75.7% and specificity was 61.0%, whereas at 0.0445 µmol/mL, the sensitivity was 70.3% and specificity was 74.0%. Thus, the choice of the final threshold should be based on the pre-specified method for determining the optimal cut-off, together with consideration of the assay’s analytical detection limits.

## 4. Discussion

Compared to matched healthy controls, we found that plasma EndoEth levels were significantly higher in traditionally abstinent Saudi adults with T2DM. This finding aligns with several previous reports. Simic and colleagues [19] and Hafez et al. [20] demonstrated that patients with T2DM or liver cirrhosis have higher EndoEth levels than controls, with the highest levels in those with both conditions. Our observation that EndoEth was detectable even during fasting indicates that hepatic clearance capacity was surpassed, a phenomenon previously noted by Cordell et al. [21] and Meijnikman et al. [5], who emphasized that persistent low-level systemic ethanol can have dire consequences on tissues, particularly the liver and pancreatic β-cells.

The significant positive association between EndoEth and disease duration (r = 0.298, *p* = 0.001) in our study supports the concept that prolonged dysbiosis and cumulative metabolic derangement enhance ethanol production. This is in line with significant increases in EndoEth upon insulin treatment (*p* = 0.001) as a late treatment option, and the positive association with TyG-IR index (and its composite fasting blood glucose and serum triglycerides) that correlated to disease progression. This is consistent with the work of Shahzad et al. [22], who reported urinary EndoEth levels reaching 6.0% in abstinent T2DM Pakistani patients with disease duration > 5 years and random blood glucose > 250 mg/dL. Similarly, Kruckenberg et al. [11] and Hafez et al. [20] noted that existing comorbidities such as diabetes amplify EndoEth generation.

We observed that patients with microvascular complications, particularly ophthalmopathy, had significantly higher EndoEth levels. This may be explained by several mechanisms. Since we had only three nephropathy patients, this finding should be taken cautiously. Shahzad et al. [22] proposed that EndoEth, together with hyperglycemic ketotic intermediary metabolism, could complicate diabetic neuropathy. Additionally, Chen et al. [23] showed that EndoEth induces mitochondrial dysfunction in non-alcoholic fatty liver disease, reducing ATP, increasing ROS, and promoting mitochondrial DNA damage—factors known to contribute to inflammation and fibrogenesis that may also affect microvascular beds. The multivariate logistic regression models found that EndoEth was significantly associated with complications when adjusted separately for either TC or LDL-C, but the association becomes nonsignificant when both closely related lipid measures were entered simultaneously. After adjustment for TC and LDL-C, each 0.01 µmol/mL increase in EndoEth (which was divided by 0.01, so the OR represents the change in odds for every 0.01 µmol/mL increase in it) was associated with approximately 6.1% higher odds of complications. This underscores the potential of EndoEth as a risk factor, in line with the suggestion of Andaloro et al. [24] that low-level but persistent microbial ethanol production may perpetuate tissue injury.

Regarding metabolic indices, EndoEth correlated significantly with insulin resistance TyG-IR index (<0.001). This mirrors the findings of Mbaye et al. [25] and Chen et al. [23], who reported that gut-derived ethanol alters hepatic lipid metabolism, increases de novo lipogenesis, and promotes triglyceride accumulation. Pai et al. [26] also noted that T2DM patients with high insulin resistance were colonized with abundant ethanol-generating microbiota. The significant association with glycemic indices reflects the multifactorial nature of glycemic control or the fact that EndoEth is closely linked to post-prandial fermentation and to long-term glycemia. The moderate and small-size effects for this relationship highlight the importance of a cautious interpretation.

Patients on insulin-containing regimens had the highest EndoEth levels, while treatment-naïve patients had undetectable levels. This could be explained by more advanced disease in insulin-treated patients, but also by the observation of Krönert et al. [27] that increased urinary alcohol was more significant in insulin-treated diabetics. Furthermore, Engstler et al. [12] demonstrated that insulin resistance alters hepatic alcohol dehydrogenase activity, potentially reducing ethanol clearance independently of the production rate. Sarkola and Eriksson [28] provided evidence that EndoEth is constantly produced in healthy men and women, detectable in the circulation, and that its rate of production increased from levels below detection limit (i.e., <5 μmol/L) to 13 ± 8 μmol/L after the intake of a sugary juice, and to 30 ± 20 μmol/L (0.138 ± 0.092 mg/dL) at 195 min after the intake of 4-methylpyrazole, a specific alcohol dehydrogenase inhibitor. In MASLD subjects, portal vein blood EndoEth is 36.9 mg/dL and in metabolic dysfunction-associated steatohepatitis (MASH) patients it is 96.8 mg/dL, reaching to more than 400 mg/dL, in the case where it is combined with gut fermentation syndrome (note that 100–150 mg/dL is the intoxication level) [6].

The moderate biomarker performance of EndoEth (AUC = 0.766, sensitivity of 75.7%, specificity of 61.0%) suggests that it may help distinguish against T2DM complications. This is comparable to the diagnostic utility of other gut-derived metabolites reported by Aragonès et al. [29] and Zhu et al. [30] in the context of metabolic dysfunction-associated steatotic liver disease. The cut-off of ≥0.016 µmol/mL determined by Youden’s index (J = 0.516) provides a practical threshold for future screening studies.

Interestingly, we did not find significant sex-dependent differences in EndoEth levels within either group, but across-group gender differences, although some case series [2] have suggested a male predominance in auto-brewery syndrome. This discrepancy may be due to population-specific differences in dietary habits, microbiome composition, or the small number of cases.

Our findings have several mechanistic implications. EndoEth can interfere with pancreatic insulin secretion, as shown by Wang et al. [31] and Yang et al. [32], who demonstrated that ethanol and acetaldehyde reduce insulin secretion in a dose-dependent manner and induce fibroblast growth factor (FGF)21 resistance-mediated β-cell dysfunction. Moreover, the induction of cytochrome P450 2E1 (CYP2E1) by ethanol [5,33] generates oxidative stress and accelerates the metabolism of many drugs, potentially complicating diabetes pharmacotherapy. The heightened NADH/NAD^+^ ratio from alcohol oxidation inhibits gluconeogenesis and fatty acid oxidation while promoting lipogenesis, as reviewed by Valenzuela et al. [34] and Stamation [35]—a pathway that may explain the association of EndoEth with dyslipidemia and insulin resistance observed in our study.

The source of EndoEth in our abstinent patients is likely multifactorial. Diabetes is a major risk factor for candiduria [36], with a prevalence of ~10% in T2DM, and Candida albicans can produce up to 1 mg/hr of ethanol per gram of intestinal content [37]. Esmailzadeh et al. [38] found strong positive correlations between candiduria, HbA1c > 7%, and glycosuria. Additionally, bacterial producers such as Klebsiella pneumoniae [39,40,41] and Enterocloster bolteae [42] have been implicated in EndoEth production. The presence of small intestinal bacterial overgrowth (SIBO), more common in diabetics [43], may further contribute to EndoEth.

Finally, while our study focused on T2DM patients without overt ABS, the stepwise increase in EndoEth from healthy controls to T2DM patients, as also noted by Hafez et al. [21] and Welch et al. [44], suggests a continuum of gut fermentation severity. This supports the concept that the persistent microbial ethanol production, even at sub-intoxicating levels, can inflict chronic damage [6,45].

The causative relationship and mechanistic explanations could not be inferred from cross-sectional design. We did not assess/implement: (1) the presence and extent of potential hepatosteatosis, (2) the more stable biomarker ethanol metabolites such as ethyl glucuronide and sulphate and phosphatidyl-ethanol, (3) the provocative carbohydrate-challenge test, (4) the narrow 2 h post-prandial EndoEth time window that reflects bacterial fermentation rather than those fermented by fungi, or an extended time-course analysis, and (5) the fact that we cannot exclude the possibility of drug/food supplement-induced inhibition of ethanol metabolizing enzymes or the inhibition of gut fermentation, and/or suppressive polymorphisms in ethanol catabolizing enzymes in our participants [2,16].

## 5. Conclusions

This study demonstrates that circulating EndoEth is significantly elevated in abstinent Saudi patients with T2DM compared to matched healthy controls. EndoEth levels positively associated with disease duration, insulin treatment, and insulin resistance (TyG-IR index), and are highest in patients with microvascular complications, particularly ophthalmopathy. Multivariable analysis models identified total and LDL-C but not EndoEth as an independent risk factor for the development of diabetic complications. The biomarker performance of EndoEth (AUC = 0.766) shows moderate ability to discriminate against T2DM complications, suggesting potential clinical utility. These findings support the emerging paradigm that microbiome dysbiosis-derived EndoEth in the setting of diabetes contributes to metabolic dysregulation, insulin resistance, and end-organ damage. The association with insulin-containing treatment and disease duration raises the possibility of a bidirectional relationship: hyperglycemia promotes ethanol-producing microbial overgrowth, and ethanol in turn impairs insulin secretion and action. EndoEth measurement may identify T2DM patients at higher risk for microvascular complications.

## Figures and Tables

**Table 1:** Baseline demographic, anthropometric and clinical characteristics of the study’s type-2 diabetic patients (125) and healthy controls (55).

Variables				n	%
Age (years)	Patients	Mean ± SD (Range)	*p* = 0.106 ^a^	56.42 ± 12.22 (23–99)	
	Controls			54.2 ± 6.00 (43–68)	
Gender	Patients	Female/Male	*p* = 0.191 ^b^	76 (60.8)/49 (39.2)	
	Controls			27 (49.1%)/28 (50.9%)	
BMI (kg/m^2^)	Patients	Mean ± SD (Range)	*p* = 0.053 ^a^	31.59 ± 7.15 (20.0–58.6)	
	Controls			29.19 ± 3.87 (19.81–43)	
Disease Duration (years)	Mean ± SD			11.01 ± 7.1	
	Median (Range)			10 (0.5–32)	
Complications	No			88	70.4%
	Yes			37	29.6%
Complication Subtypes	Ophthalmopathy			21	16.8%
	Neuropathy			15	12.0%
	Nephropathy			3	2.4%
	Myocardial Infarction			1	0.8%
	Stroke			2	1.6%
Treatment Type	Naïve/Diet Control			8	6.4%
	Metformin			49	39.2%
	Hypoglycemics ± Metformin			22	17.6%
	Insulin-containing regimen			46	36.8%

Data shown are mean ± SD (range) or frequencies [n (%)], and *p* value of independent *t*-test ^a^ or chi-squared test ^b^. BMI = body mass index.

**Table 2:** Comparison of the metabolic and hematological parameters between T2DM patients and healthy controls.

Variables	Controls (n = 55)	Patients (n = 125)	*p* Value
	Mean ± SD or Median ± IQR (Range)	Mean ± SD or Median ± IQR (Range)	
HbA1c (%)	5.5 ± 0.37	8.03 ± 1.57	<0.001 ^a^
FBG (mM/L)	5.03 ± 0.34	8.69 ± 3.18	<0.001 ^a^
TG (mM/L)	1.08 ± 0.31	1.67 ± 0.79	<0.001 ^a^
TyG-IR index	8.33 ± 0.32	9.18 ± 0.99	<0.001 ^a^
TC (mM/L)	4.79 ± 0.62	5.05 ± 1.01	0.043 ^a^
HDL-C (mM/L)	1.74 ± 0.13	1.27 ± 0.28	<0.001 ^a^
LDL-C (mM/L)	2.04 ± 0.36	2.53 ± 1.09	<0.001 ^a^
WBCs (×1000/µL)	7.01 ± 2.12	7.8 ± 2.20	0.035 ^a^
Endogenous ethanol (µmol/mL)	0.00 ± 0.00 (0.0–0.27)	0.021 ± 0.11 (0.0–0.32)	<0.001 ^b^

Data shown are mean ± SD or median ± IQR (range) and *p* value of the independent *t*-test ^a^ or Mann–Whitney U test ^b^. FBG = fasting blood glucose. HbA1c = hemoglobin A1_C_. TyG-IR = triglycerides-glucose insulin resistance. TC = total cholesterol. HDL-C = high density lipoprotein cholesterol. LDL-C = low density lipoprotein cholesterol. TG = plasma triglycerides. WBCs = white blood cells.

**Table 3:** Subgroup analysis of endogenous ethanol levels in T2DM patients stratified by demographic and clinical characteristics.

Variables		Endogenous Ethanol (µmol/mL)		*p* Value
		Median	IQR	
Gender	Female (n = 76)	0.002	0.060	0.515 ^a^
	Male (n = 49)	0.002	0.080	
Complications	No (n = 88)	0.011	0.050	<0.001 ^a^
	Yes (n = 37)	0.105	0.150	
Ophthalmopathy	No (n = 104)	0.011	0.070	0.001 ^a^
	Yes (n = 21)	0.109	0.160	
Nephropathy	No (n = 122)	0.011	0.090	<0.001 ^a^
	Yes (n = 3)	0.169	- *	
Neuropathy	No (n = 110)	0.011	0.100	0.106 ^a^
	Yes (n = 15)	0.052	0.130	
Treatment Type	Naïve/Diet Control (n = 8)	0.000	0.000	0.001 ^b^
	Metformin (n = 49)	0.016	0.080	
	Hypoglycemics ± Metformin (n = 22)	0.002	0.100	
	Insulin-containing regimen (n = 46)	0.079	0.160	

Data shown are median ± IQR and *p* value of Mann–Whitney U test ^a^ or Kruskal–Wallis test ^b^. * As the values are constant among all the three observations.

**Table 4:** Spearman correlation analysis of endogenous ethanol levels with the demographic and biochemical parameters in T2DM patients.

Endogenous Ethanol (µmol/mL) Versus	Spearman Correlation	Effect Size	*p* Value
Age (years)	0.153	Small	0.107
BMI (kg/m^2^)	−0.147	Small	0.128
Disease Duration (years)	0.298	Moderate	0.001
HbA1c (%)	0.150	Small	0.112
FBG (mM/L)	0.193	Small	0.04
TG (mM/L)	0.303	Moderate	0.001
TyG-IR index	0.274	Moderate	0.003
TC (mM/L)	0.180	Small	0.069
HDL-C (mM/L)	0.180	Small	0.072
LDL-C (mM/L)	−0.061	Negligible	0.568
WBCs (×1000/μL)	−0.044	Negligible	0.682

Data shown are r and *p* values and effect size. Refer to the footnote of Table 2 for definition of abbreviations.

**Table 5:** Comparison of demographic and clinical characteristics between T2DM patients with and without complications.

Variables		Complications				*p* Value
		No (n = 88)		Yes (n = 37)		
		n	%	n	%	
Age (years)	Mean ± SD	54.88 ± 12.66		60.0 ± 12.42		0.033 ^a^
BMI (k/m^2^)	Mean ± SD	32.13 ± 7.68		30.37 ± 5.68		0.215 ^a^
Gender	Female	60	78.9%	16	21.1%	0.015 ^b^
	Male	28	57.1%	21	42.9%	
Disease Duration (years)	Mean ± SD	9.12 ± 5.99		15.51 ± 7.55		<0.001 ^a^
Disease Duration (years)	≤3	15	100.0%	0	0.0%	0.005 ^b^
	>3–5	13	86.7%	2	13.3%	
	>5	60	63.2%	35	36.8%	
Disease Duration (years)	≤10	61	81.3%	14	18.7%	0.001 ^b^
	>10	27	54.0%	23	46.0%	
HbA1c (%)	Mean ± SD	7.88 ± 1.44		8.38 ± 1.80		0.098
FBG (mM/L)	Mean ± SD	8.20 ± 2.66		9.85 ± 3.98		0.008 ^a^
TG (mM/L)	Mean ± SD	1.65 ± 0.69		1.73 ± 0.99		0.594
TyG-IR Index	Mean ± SD	9.12 ± 1.07		9.33 ± 0.73		0.279
TC (mM/L)	Mean ± SD	4.85 ± 1.07		5.59 ± 0.56		<0.001 ^a^
HDL-C (mM/L)	Mean ± SD	1.29 ± 0.27		1.22 ± 0.30		0.265
LDL-C (mM/L)	Mean ± SD	2.22 ± 0.72		2.65 ± 1.2		0.033 ^a^
WBCs (×1000/μL)	Mean ± SD	7.9 ± 1.98		7.6 2 ± 2.62		0.562
Endogenous Ethanol (µmol /mL)	Median ± IQR	0.011 ± 0.05		0.105 ± 0.15		<0.001 ^c^

Data shown are Mean ± SD, frequencies [n/%], or Median ± IQR and *p* values of independent *t*-test ^a^, chi-squared test ^b^, or Mann–Whitney U test ^c^.

**Table 6:** Multivariable logistic regression model for the development of diabetic complications.

Complications Versus	B	S.E.	Wald	df	*p* Value	OR	95% CI for OR	
							Lower	Upper
Endogenous ethanol (per 0.01 µmol/mL)	0.059	0.040	2.156	1	0.142	1.061	0.980	1.148
LDL-C	−1.641	0.459	12.767	1	<0.001	0.194	0.079	0.477
TC	1.871	0.504	13.775	1	<0.001	6.496	2.418	17.451

Data shown are output statistics for significant predictors. Refer to the footnote of Table 2 for definition of abbreviations.

## Data Availability

The major data supporting the findings of this study are presented in the article and the spreadsheet of the raw data is available from the corresponding authors upon reasonable request and after proper local ethical approval.

## Abbreviations

The following abbreviations are used in this manuscript:

ATP	Adenosine triphosphate
AUC	Area under the Receiver Operating Characteristic (ROC) curve
BMI	Body mass index
CI	Confidence interval
CYP2E1	Cytochrome P450 2E1
CV	Coefficient of variation
DNA	Deoxyribonucleic acid
EDTA	Ethylenediaminetetraacetic acid
EndoEth	Endogenous ethanol
FBG	Fasting blood glucose
FGF21	Fibroblast growth factor
HbA1c	Glycated hemoglobin A1c
HDL-C	High-density lipoprotein cholesterol
LDL-C	Low-density lipoprotein cholesterol
MASLD	Metabolic dysfunction-associated steatotic liver disease
NAD^+^	Nicotinamide adenine dinucleotide
NADH	Reduced nicotinamide adenine dinucleotide
NAFLD	Non-alcoholic fatty liver disease
SD	Standard deviation from the mean
SIBO	Small intestinal bacterial overgrowth
OR	Odds ratio
ROS	Reactive oxygen species
T1DM	Type-1 diabetes mellitus
T2-DM	Type-2 diabetes mellitus
TG	Triglycerides
TC	Total cholesterol
TyG-IR	Triglyceride–glucose insulin resistance
WBCs	Total leukocyte count
